# A survey of cone beam computed tomography use amongst endodontic specialists in the United Kingdom

**DOI:** 10.1111/iej.14203

**Published:** 2025-02-18

**Authors:** Shanon Patel, Jackie Brown, Federico Foschi, Nassr Al‐Nuaimi, James Fitton

**Affiliations:** ^1^ Faculty of Dentistry, Oral & Craniofacial Sciences King's College London London UK; ^2^ Guy's & St Thomas NHS Foundation Trust London UK; ^3^ Private Practice London UK; ^4^ Eastman Dental Institute, University College London London UK; ^5^ Boston University Henry M. Goldman School of Dental Medicine Boston Massachusetts USA; ^6^ College of Dentistry University of Baghdad Baghdad Iraq

**Keywords:** CBCT, endodontics, selection criteria

## Abstract

**Introduction:**

The aim of this study was to investigate the applications of cone beam computed tomography (CBCT) amongst endodontic specialists in the United Kingdom (UK) via an online survey.

**Methods:**

An online invitation to take part in the survey was sent out to 306 specialist endodontists registered on the UK specialist register. The survey consisted of a series of questions associated with demographics, access and use of CBCT, utilization of dose optimization parameters, and frequency of use in different clinical scenarios.

**Results:**

In total, 202 respondents completed the survey (a response rate of 66.3%), 128 were male (63.4%), and 74 were female (36.6%). Of the 202 respondents, 174 (85.7%) used CBCT for diagnosis and/or management of endodontic problems. A CBCT scanner was on‐site in 71.3% (*n* = 124) and 28.7% (*n* = 50) being off‐site. A small field of view (FOV) was the prescribed scan in most cases (88.5%, *n* = 154/174). The cost of the CBCT scan was included in the assessment/treatment fee in 21.8% (38/174) of cases, with 78.2% (136/174) charged a separate fee for the scan. In total, 89.1% (155/174) respondents used CBCT ‘often or always’ for management of complex root canal anatomy, 87.4% (152/174) for root resorption, 84.5% (147/174) for periapical microsurgery, only 20.7% (36/174) of respondents would use CBCT to assess the outcome of treatment. Only 35.1% (61/174) of respondents would prescribe a CBCT scan for a pregnant patient and 78.2% (136/174) would take a CBCT scan for a paediatric patient. In total, 22.4% (39/174) of respondents did not report or request reports of their CBCT scans. Respondents chose to alter the exposure parameters depending on the patient's age or if the dentition was deemed extensively restored. Of the clinicians who incorporate CBCT into their practice, 93.7% (164/174) believe it improves the quality of the care they provide, and 93.6% (163/174) felt that the use of CBCT improves confidence in their diagnosis.

**Conclusions:**

The use of CBCT amongst specialist endodontists in the UK is commonplace. However, usage does not appear to completely follow best practice guidance for certain clinical scenarios and highlights the need for further training in CBCT.

## INTRODUCTION

Radiography is a cornerstone for the diagnosis and management of endodontic problems; however, it does have limitations which include anatomical noise, geometric distortion and the two‐dimensional nature of the image produced. These limitations can hinder accurate assessment of teeth and the adjacent anatomy which may have an impact on the diagnosis and/or management of the endodontic problem (Krug et al., 2019; Patel et al., 2019).

Cone beam computed tomography (CBCT) overcomes these limitations by generating reconstructed images of the region of interest in three orthogonal planes. CBCT has been shown to improve the assessment of complex endodontic problems including internal inflammatory and external cervical resorption, complex root canal anatomy, perforations, and root fractures (de Souza et al., 2017; Ee et al., 2014; Patel et al., 2021). Furthermore, the diagnostic information using CBCT frequently results in a change or modification of the treatment plan chosen (Mazon et al., 2022; Rodriguez et al., 2017), as well as improving confidence in their decision‐making (Patel et al., 2021; Wanzeler et al., 2020).

The impact of CBCT in the diagnosis and management of endodontic problems is reflected by position statements being published by professional organizations (American Association Endodontists/American Academy of Maxillofacial Radiology, 2015; Patel et al., 2019). These guidelines highlight that radiographs should be used in the first instance, and when indicated, that is, in complex cases, a CBCT may be recommended. In endodontics, a small field of view (FOV) scan is recommended, thus minimizing radiation dose. A CBCT scan must be justified on a case‐by‐case basis, patients must be made aware of increased radiation exposure as well as any additional fees associated with the acquisition and/or reporting of the scan. The image quality and diagnostic yield vary between scanners, furthermore, the presence of artefacts associated with radiodense objects in the region of interest as well as poor resolution compared with conventional radiography may have an impact on the quality of the image generated (Patel et al., 2019). It is recommended that the exposure parameters should be tailored to the specific endodontic task (ALARA), considering the patient's age and potential artefact from neighbouring radiodense objects (Faculty of General Dental Practitioners, 2020; Farman & Farman, 2005; SEDENTEXCT, 2012).

A web‐based survey assessed the use of CBCT amongst endodontists in the USA found that 93% of clinicians had access to CBCT in their residency (specialist training) programmes compared with 82% of endodontists who had been practicing for less than 10 years, and 78% of endodontists practicing for more than 11 years (Setzer et al., 2017). Over 45% of respondents ‘frequently’ or ‘always’ took a CBCT for managing internal/external resorption and prior to periapical microsurgery. There was less use of CBCT for nonsurgical retreatments (24.9%), differential diagnosis (21.2%), and identifying periradicular lesions (18.3%). A survey of 742 Brazilian endodontists found that CBCT was used for assessment of suspected vertical root fractures (64%), locating canals (58.7%), perforations (53.9%), and root resorption (42.1%) (Paiva et al., 2023). A survey of 117 German and Swiss Endodontists concluded that CBCT was valuable for managing highly complex endodontic cases (Krug et al., 2019). These types of surveys highlight areas which need more focus in basic and/or specialist training, as well as continuing professional development courses (CPD). An insight into use of CBCT amongst UK endodontists would be desirable to assess not only if they follow current ESE guidelines when deciding on taking a CBCT, but crucially to assess if they tailor the exposure parameters according to application as well as radiosensitivity of the patient. Furthermore, reporting on CBCT scans is an essential part of the CBCT workflow, however, to date, compliance on this mandatory task has not been assessed.

The aim of this study was to investigate the uptake of CBCT amongst UK endodontists, their demographics, and indications for use.

## METHODS

This study followed STROBE checklist (Von Elm et al., 2014) and ethical approval was sought from the Research Ethics Committee, King's College London (KCL). Ethical clearance was granted (MSRU‐21/22–29 591).

A list of UK‐registered endodontists was collected from the General Dental Council (GDC) specialist list (www.GDC.org). A link to an online survey (Typeform, Barcelona, Spain) was sent by email to these registrants. The email informed the respondents that the survey was part of a research project in the KCL Faculty of Dentistry, Oral & Craniofacial Sciences, Guy's Hospital, London. No identifiable information was requested from the participants to allow for unbiased responses.

The survey consisted of a series of questions (Table 1), which included the respondents' demographics, use and accessibility of CBCT, tailoring of the exposure parameters to the patient's specific clinical scenario, and the frequency of use of CBCT in different clinical scenarios. The participants were asked if they felt taking a CBCT improved the quality of care they provide, confidence in their diagnosis, and patient's attitudes towards having a scan taken.

**TABLE 1:** The survey form.

Survey questions
What is your age?
What is your gender?
What is your work/practice setting? Practice only Hospital setting only Practice and hospital/university setting Other
How much of your clinical time is dedicated to endo? Up to 25% 25%–50% 50%–75% 75+%
Where did you complete your specialist training?
How long ago did you finish your endodontic specialist training? 0–4 years 5–10 years 11–15 years 16–20 years 21+ years
Do you use CBCT for endodontic assessment? Yes No
How long have you been using CBCT? 0–5 years 6–10 years 11–15 years 16+ years
Is your CBCT scanner On‐site? Off‐site/referral to a different practice/setting?
What field of view (FOV/volume) size do you use? Small FOV (<5 cm) Medium FOV (5–10 cm volume, limited to single arch) Large FOV (>10 cm volume, full facial region) Unsure
What features do you look for in a scanner? Image quality Low ionizing radiation dose Financial cost Local availability Combination of the above Variety of exposure settings Other
Fee for CBCT scan is: Included in overall treatment fee Not included Please specify fee to patients including any report of the scan
Do you report yourself on CBCT scans? Yes No Occasionally
If you do not report on the CBCT scan who provides the report? Specialist dental/oral and maxillofacial radiologist Colleague with appropriate training for reporting on scans Scans are not formally reported on
If you do not feel comfortable reporting on a scan, in which circumstances would you refer to a colleague to report on the scan?
How often do you report on a scan in the following circumstances (always, often, sometimes, rarely, never): Clinical/radiographic apical pathosis in inconclusive Assessment and treatment of dento‐alveolar trauma Complex root canal anatomy (Dens invaginatus) Complex nonsurgical retreatment cases (untreated canals/perforations) Assessment and management of root resorption Pre‐operative assessment for periradicular surgery Identification of obliterated root canals Detection of periradicular bone changes indicative of root fractures Assessment of healing/outcome of endodontic treatment Proximity of vital adjacent anatomy (e.g. maxillary sinus/ID canal) to apex of the treatment tooth
Would you feel comfortable prescribing a CBCT scan for a pregnant patient? Yes No
(If no) Why would you no feel comfortable prescribing a CBCT scan for a pregnant patient?
Would you feel confident prescribing a CBCT scan for a paediatric patient (less than 18 years old)? Yes No
(If no) Why would you not feel comfortable prescribing a CBCT scan for a paediatric patient (less than 18 years old)?
When prescribing a CBCT scan, do you consider altering any of the following parameters (more than one box can be checked)? Tube current (mA) Tube voltage (kV) Exposure time (s) Voxel size/image resolution Field of view (FOV) Rotation length (°)
In which circumstances might you change image parameters? Young patient (<25 years old) Extensively restored dentition (e.g. implants, post‐restorations) Other
Do you ever take mid‐treatment scans? Yes No
(If yes) In which situation would you take a mid‐treatment scan?
Do you ever take post‐treatment scans? Yes No
(If yes) In which situation would you take a post‐treatment scan?
Do you feel taking a CBCT improves the quality of care you provide? Yes No
Do you feel taking a CBCT improves confidence in your diagnosis? Yes No

Chi‐squared test was used to compare the homogeneity of proportions of categories of answers through levels of a second variable. Fisher's exact test was conducted if the frequencies of some categories were too low. Mann–Whitney's test was used to compare distributions of ordinal or continuous variables through two independent groups of respondents. Spearman's correlation was used to assess the non‐linear association between variables measured on an ordinal scale. The reference level of significance was set up to 5% (*α* = 0.05).

A sample size calculation was carried out using G*Power software (version 3.1.9.6, Franz Faul; Christian‐Albrechts‐Universität Kiel). A sample of *n* = 202 would provide a maximum error of 6.8% to estimate a proportion of total population assuming 95% of confidence and *p* = *q* = 0.5 for that proportion. In addition, 82.8 of statistical power is reached to detect differences between proportions 30%–50% between the two groups as significant. Descriptive statistics were carried out with SPSS software for Mac (version 28; SPSS Inc., IBM, New York, NY, USA) to assess data distribution.

## RESULTS

In total 202 of 306 respondents contacted completed the questionnaire: resulting in a response with a 66.3% completion rate. Of these, 128 were male (63.4%) and 74 were female (36.6%). The age group distribution was observed as <30 years (9.9% [*n* = 20]), 31–40 years (40.6%, [*n* = 82]), 41–50 years (34.2% [*n* = 69]), 51–61 years (14.4% [*n* = 29], and 61+ years, 1% [*n* = 2]). A total of 48.5% (*n* = 98) worked exclusively in specialist practice, 43.6% (*n* = 88) worked in both practice and university settings, and 7.9% (*n* = 16) exclusively in university.

The majority (86.1%, *n* = 174/202) of the respondents used CBCT, 95.5% of the respondents who used CBCT worked in both practice and university settings, compared with 81.3% and 78.6% of respondents who worked exclusively in specialist practice and university settings, respectively (*p* = .003). A total of 71.8% (125/174) of respondents had access to an on‐site CBCT, and 28.2% (49/174) referred patients to an off‐site for a CBCT scan. Of those who used CBCT, 88.5% (154/174) had access to a small FOV scans, 4.6% (8/174) and 1.7% (3/174) used medium and large FOV, respectively.

When deciding on prescribing a CBCT scan the most important features were; image quality 75.9% (132/174), radiation dose 44.3% (77/174), choice of exposure settings 24.7%, scanner cost 23% (40/174), availability 21.3% (37/174), and combination of features 39.7% (69/174).

The CBCT scan was charged as a separate fee from the endodontic treatment in 78.2% (136/174) of cases, the remainder of the respondents included the CBCT scan in the overall consultation fee (21.8%, *n* = 38/174).

In total, when indicated 35.1% (61/174) of respondents would prescribe a CBCT scan for a pregnant patient and 78.2% (136/174) would take a CBCT scan for a paediatric (adolescent) patient.

Of the respondents using CBCT, 93.7% (163/174) felt CBCT improved their confidence in diagnosis, and 94.3% (164/174) felt that CBCT improved the quality of care they provided for patients.

**FIGURE 1:**
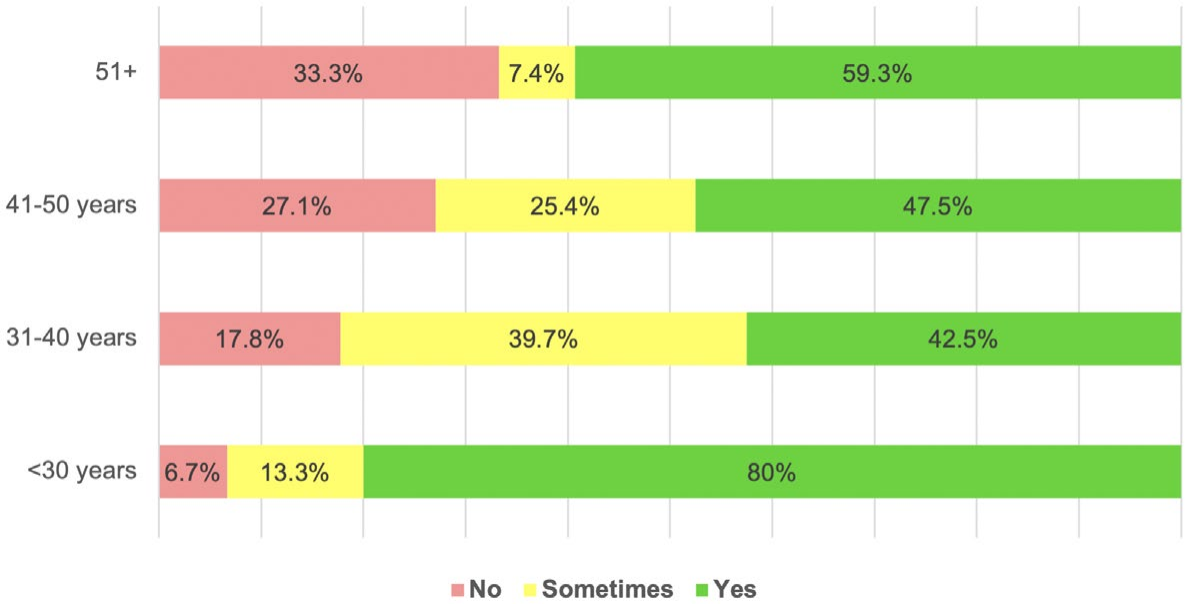
Percentage of writing a CBCT reporting by respondent age.

In total, 22.4% (39/174) of respondents did not report or request radiographic reports. The prevalence of not reporting on CBCT increased progressively with age, with 6.7% (1/15) not reporting in the <30‐year‐old age group to 33.3% not reporting in the 51+ year age group (*n* = 9/27, *p* = .008) (Figure 1).

The respondents would consider adjusting the voxel size 44.3% (77/174), tube current 25.3% (44/174), exposure time 23% (40/174), rotation arc 16.1% (28/174), and tube voltage 15.5% (27/174) to maximize the diagnostic yield.

Most respondents had used CBCT for up to 5 years (60.9%, *n* = 106/174). The length of time using CBCT increased with the respondent's age (*p* = .018), 19.8% (17/86) of the respondent's aged more than 40 years have been using CBCT for more than 10 years compared with only 5.7% (5/88) of those aged less than 40 years.

There was a significant association between prescribing a CBCT scan for a pregnant patient and the age of the respondent (*p* = .004). Respondents aged <30 years were the most comfortable prescribing scans for pregnant patients (66.7%, 10/15), whilst respondents aged 31–40 years were the least comfortable prescribing scans (21.9%, *n* = 16/73).

**FIGURE 2:**
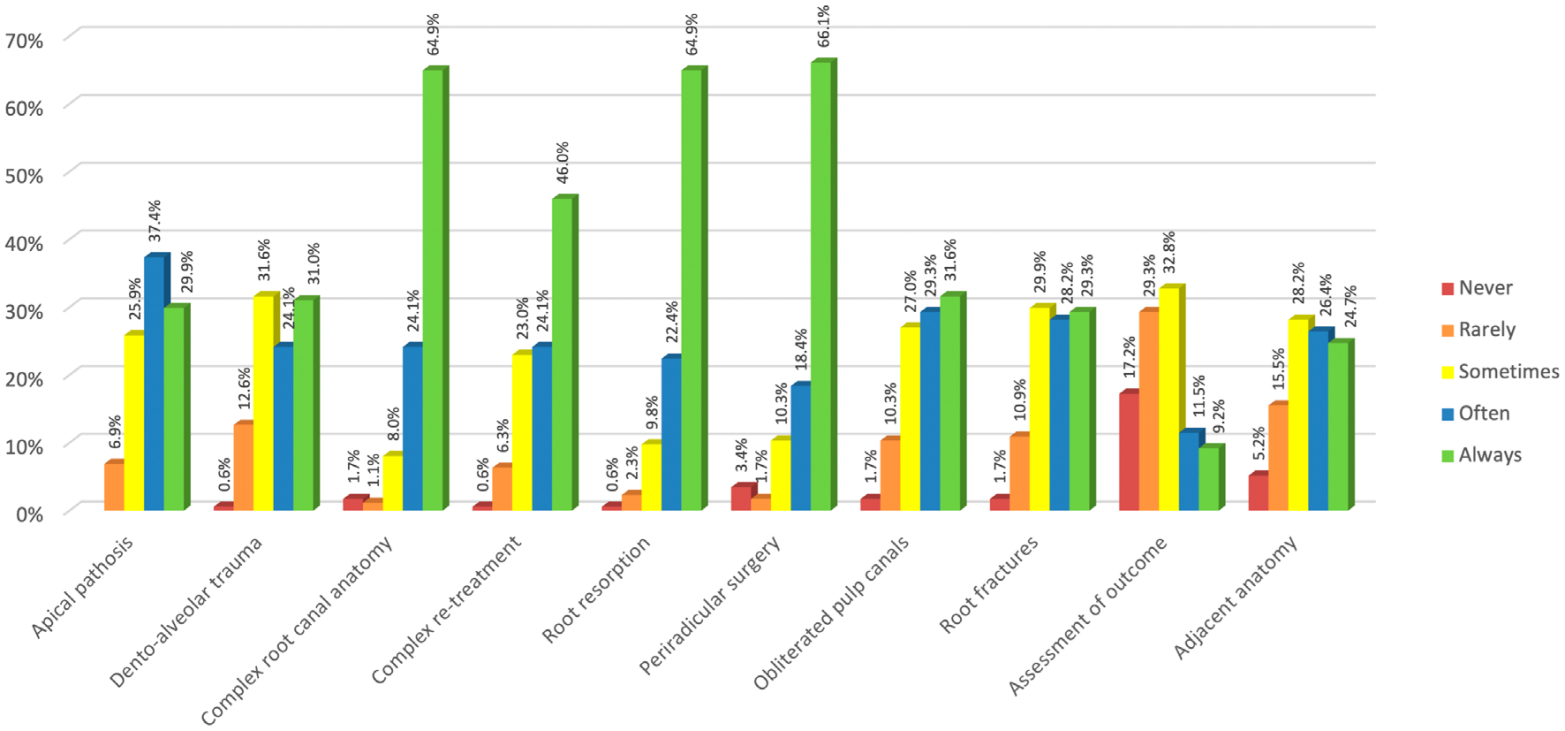
Frequency of prescribing CBCT for different clinical indications.

In total, 89.1% (155/174) of respondents used CBCT ‘often or always’ for the management of complex root canal anatomy, 87.4% (152/174) for root resorption, 84.5% (147/174) for periapical microsurgery, the least common reason prescribing CBCT was to assess the outcome of treatment, with only 20.7% (36/174) of respondents saying they ‘often/always’ used CBCT for this clinical indication (Figure 2).

When indicated, 55.7% (97/174) of respondents took mid‐treatment scans and 28.2% (49/174) post‐treatment scans. Younger endodontists (<30 years, 80%, *n* = 12/15) were more likely to use CBCT for managing complex root anatomy, compared with endodontists aged 41–50 years (49.2%, *n* = 29/59) and endodontists aged 51+ years (63%, *n* = 17/27) (*p* = .011).

**FIGURE 3:**
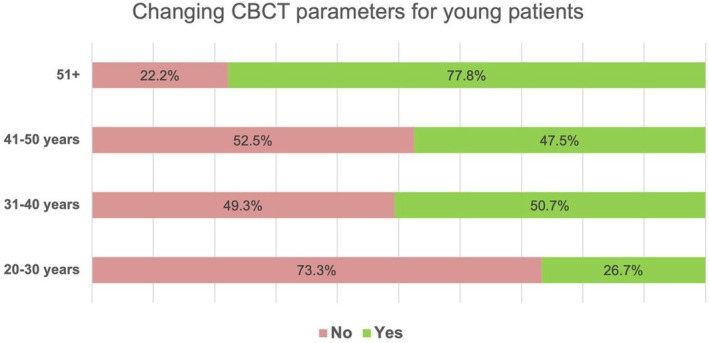
Percentage of changing CBCT parameters for young patients.

Furthermore, younger endodontists (<30 years, 73.3%, *n* = 11/15) were most hesitant to change exposure parameters for young patients compared with older respondents (51+ years, 22.2%, *n* = 6/27) (*p* = .009) (Figure 3).

Differences in gender were only significant for the frequency of prescribing a scan in a case of adjacent vital anatomy to the apex of a tooth under treatment as 35% (21/60) of females always prescribed versus 19.3% (22/114) of male respondents (*p* = .025).

Clinicians with on‐site access were more likely to include the CBCT fee in the overall consultation fee (26.4%, *n* = 33/125, *p* = .020) versus off‐site access (10.2%, *n* = 5/49).

## DISCUSSION

To the authors' knowledge this is the first study to assess the prescribing patterns amongst most UK‐registered specialist endodontists. A global survey of 543 endodontists and endodontic residents found that 91.2% used CBCT, this study included respondents from the UK who were grouped in a ‘Europe/Middle East’ group (Cheung et al., 2023). A survey of the use of CBCT amongst UK‐registered specialist endodontists was desirable to assess if they were tailoring the exposure parameters to the clinical need and following recommended guidelines (Patel et al., 2019; SEDENTEXCT, 2012).

The impact of additional information a CBCT offers over PA is well established. A 35%–55% change in diagnosis has been reported when cases first assessed with PA were reassessed later with CBCT (Chogle et al., 2020; Mota et al., 2015). Furthermore, it has been shown that the additional information from CBCT results in a change of treatment plan compared with when cases are assessed only with a PA in 27% to 62% of cases assessed (Chogle et al., 2020; Ee et al., 2014; Rodriguez et al., 2017).

The questions in the survey were designed to encompass the selection criteria for the use of CBCT in endodontics as well as to assess if clinicians were adjusting the exposure parameters to tailor the scan to their patients and the diagnostic information required (ALARA) published in the European Society of Endodontology's (ESE) position statement on CBCT (Patel et al., 2019; SEDENTEXCT, 2012).

An in‐person survey may have yielded a higher response rate; however, this was not practical as the respondents were based all over the UK and therefore it would have been expensive and time‐consuming to do (Banning et al., 2021). Online questionnaires have the advantage of being rapid and a cost‐efficient method for the collection of data, in addition, the respondents can complete them in their own time (Jones et al., 2013; Sax et al., 2003). This online survey gave an invaluable insight into the use of CBCT amongst endodontists working in different settings across the UK.

Of the 306 endodontists listed on the General Dental Council (2020) specialist register, 203 responded, giving a response rate of 66.3%. This compares favourably to similar online CBCT surveys carried out in the USA and Germany/Switzerland which had response rates of 35.3% (1083/3076 invitees) and 51.4% (95/195 invitees), respectively (Krug et al., 2019; Setzer et al., 2017). A recent systematic review reported an average response rate of 51% for online questionnaires amongst healthcare professionals (Meyer et al., 2022). The relatively high recall in this study may reflect respondents perceiving this area of endodontics as relevant to their clinical practice and therefore completing the survey. As the questionnaire was anonymous, it was not possible to send a follow‐up email to non‐respondents, this also meant that the profile of non‐responders could not be analysed.

Most respondents (85.7%, *n* = 174) used CBCT, this was in the same order of magnitude as a similar online survey in the USA which found that 80.3% of respondents using CBCT (Setzer et al., 2017). A survey of German and Swiss Endodontists found that 53% and 32% respectively, used CBCT for diagnostic and management of endodontic problems, the lower uptake may be in part due to the majority of CBCT scanners being located in off‐site imaging centres (Krug et al., 2019). In the present study, the uptake of CBCT was higher amongst those who worked in both practice and university compared with those who worked exclusively in either private practice or university settings. More research is required to determine what lies behind these differences in prescribing CBCT amongst endodontist working in different settings.

Most respondents had access to an on‐site scanner (71.3%, *n* = 124) compared with 28.7% (*n* = 50) who referred off‐site for a CBCT scan. A similar study in the USA amongst endodontists found that the ratio of on‐site to off‐site scanners was 50.7% to 49.3% (Setzer et al., 2017). The high number of on‐site scanners in the present study reflects the importance endodontists perceive CBCT to be for the management of their patients. The increased uptake of CBCT scanners may also be due increased availability of CBCT scanners and the relative hardware costs reducing over the last 15 years.

The frequency of CBCT scans prescribed was related to whether the CBCT scanner was on‐site or off‐site, the ratio of taking scans on‐site to off‐site was 2.5 to 1. The increased uptake on CBCT use when the scanner was on‐site indicated that when access for scans was convenient there was a greater willingness to prescribe a scan as this meant the scan could be assessed and any relevant findings related to the diagnosis and/or management could be discussed with the patient in the same session. In addition, the patient may also find it more convenient. Whereas referral for a CBCT scan off‐site meant that a second consultation would be required which patients and/or the treating clinician may have felt was an inconvenience as it may delay diagnosis and treatment planning (Setzer et al., 2017). The fee for the CBCT scan was more commonly included in the overall treatment fee for on‐site scans (*p* = .020). If referred off‐site this would result in increased cost to the patient and/or a delay in completion of their treatment and increased time spent in the dental office, these factors may also dissuade endodontists from prescribing a CBCT scan off‐site.

In total, 71.3% (125/174) of respondents would, when indicated prescribe an intra‐operative scan, this compares to 44.7% of endodontists taking intra‐operative scans reported by Krug et al. (2019). However, it is not clear in either investigation if pre‐treatment CBCT scans were taken or not. If a pre‐treatment had not been taken in the first instance, then taking an intra‐operative scan would appear to be logical if during treatment more diagnostic information was required, for example, managing an ECR defect which appears on exposure to be more extensive than original thought based on the clinical examination and PA.

The results of this survey revealed that most respondents frequently/always used CBCT for the management of complex endodontic issues; 89.1% for complex root anatomy, 87.4% for assessment and management of root resorption, 84.5% for assessment for periradicular surgery; and 70.1% for the management of complex nonsurgical retreatment cases; the high uptake in these treatment categories reflects the complexity of managing these types of cases. The clinical situations in the current survey reflected the clinical scenarios published in ESE CBCT position statement on CBCT (Patel et al., 2019). UK endodontists appeared to use CBCT less frequently for dental trauma (55.2%) and obliterated canals (60.9%) when compared with endodontists globally who used CBCT more often in these clinical scenarios; dental trauma 73.9% and obliterated canals 71.9%, respectively (Cheung et al., 2023).

Endodontists under the age of 30 were more likely to prescribe a CBCT for managing complex root anatomy, compared with endodontists aged 41+ years (*p* = .011). This may reflect increased experience and understanding of root canal anatomy which comes with increased clinical experience and reflective practice resulting in marginally less scans being taken in the latter group (Campbell & Rogers, 2022; Gulabivala & Ng, 2023). Another explanation may be that CBCT has been incorporated in specialist training over the two decades meaning younger endodontists were specifically trained to use it for these clinical scenarios.

As with any ionizing radiation technique, the potential benefit of CBCT must be balanced with the increased ionizing radiation exposure and follow the ALARA (as low as reasonably achievable) principle. The ESE CBCT position statement and the American Association Endodontists/American Academy of Maxillofacial Radiology (AAE/AAOMR) Joint Position Statement highlight that each exposure should be customized for each patient's individual needs, rather than assuming that the manufacturer's default settings are most appropriate (AAE, 2016; Patel et al., 2019). This is especially relevant with children and young adults who are more radiosensitive. Pauwels et al. (2015) found that although noise increased with lower mA settings the image produced often was adequate at exposure levels which were lower than the manufacturer's recommended settings. Goulston et al. (2016) carried out a systematic review of the literature on dose optimization by altering exposure parameters and concluded that it was possible to optimize exposure parameters and therefore reduce dose whilst maintaining acceptable image quality.

On searching scientific databases, for example, Scopus, PubMed, Web of Science, ERIC, ScienceDirect, Directory of Open Access Journals it appears that this is the first time that endodontist's views on adjusting CBCT exposure parameters were assessed. The voxel size was the most adjusted (44.3%), most respondents did not alter dose parameters, with only 25.3% altering tube current, 15.5% altering tube voltage, and 23% and 16.1% changing exposure time and rotation length, respectively. More training in this area is required for endodontists to ensure they follow the ALARA principle and tailor the scan for each patient depending on age and the specific endodontic problem being assessed. In the present study, younger endodontists (<30 years) were most hesitant to change parameters for paediatric patients (<18 years old) compared with older professionals (51+ years) (*p* = .009). This may be due to a lack of understanding, training, and/or confidence in changing exposure parameters.

In total, 94.8% (163/172) of respondents felt CBCT improved their confidence in diagnosis, and 95.2% (164/172) felt that CBCT improved the quality of care they provided for patients. Wanzeler et al. (2020) concluded that CBCT increased endodontist's confidence to diagnose and manage endodontic problems when compared with PA, a similar conclusion was reported by Mota de Almeida et al. (2014). These results were self‐reported levels of confidence as it was not possible to carry out an objective analysis of this self‐perceived data. Subjective measurements could have been confirmed through an audit or reassessing the clinical cases in the presence and absence of CBCT imaging, however, this was beyond the scope of a survey.

A limitation of this online questionnaire study was that the results were based on a variety of generic endodontic problems rather than specific cases which may have permitted more objective, targeted evaluation. However, a more detailed evaluation would have increased the number of questions and time taken to complete the questionnaire which would have resulted in respondent fatigue and/or increase the likelihood of questionnaires not being completed.

As well as managing more challenging cases, increased confidence also has other benefits for the clinician such as an appreciation of their limitations as well as improved job satisfaction (Fine et al., 2018).

It is essential to assess and report on the entire CBCT scan (Brown et al., 2014). The aim of a CBCT report is to provide an accurate interpretation of the images being assessed (Patel & Harvey, 2021). The present study found that 22.4% (39/174) were not reported upon; older endodontists (41+ years) were more likely not to report (31.4%) on scans compared with younger endodontists (<40 years) (15.9%). Radiographic reports are an essential part of the patient's clinical records, this formal documentation provides evidence that X‐ray images have been reviewed, facilitates continuing care, and minimizes the likelihood of another clinician repeating the same image (Kiu et al., 2010). In the UK, it is required by law that every medical exposure should duly be reported (evaluated) and documented (Ionizing Radiation [Medical Exposure] Regulations (IRMER) 2017). To the author's knowledge, this is the first study that has specifically assessed the incidence of reporting on CBCT scans. The lack of compliance in 22.4% of clinicians may be due to a lack of awareness of current legislation and indicate that clinical audits are required to ensure best practice is being followed.

Paediatric patients have a greater risk of the stochastic effect of ionizing radiation (Isaacson et al., 2015). One aspect of this study was to gain insight into endodontist's use of CBCT in young patients, 21.8% of respondents did not take scans for paediatric patients, written responses included, ‘they are more radio‐sensitive’, ‘concerns regarding radiation to growing child’. Several respondents admitted to being unfamiliar with guidelines relating to radiation dose and paediatric patients. The European Archives of Paediatric Dentistry provides guidance on the use of CBCT in children, their recommendations include low‐dose settings, shorter exposure times, and lower mA and kV settings. These changes should be made on an individual basis ensuring that a diagnostically acceptable image is still produced (Kühnisch et al., 2020); several studies have demonstrated that reducing the exposure settings still results in diagnostically accurate CBCT images (Durack et al., 2011; Jones et al., 2015).

Over the last 50 years, there have been significant developments in X‐ray imaging resulting in reductions in ionizing doses and exposure (Flagler et al., 2022). The foetal dose even without lead shielding in reported to be less than 1% of the annual dose limit (Kelaranta et al., 2016). However, in total, only 35.1% of respondents would consider taking CBCT scans for pregnant patients, with younger respondents (<30 years) being more comfortable prescribing scans for pregnant patients. The 64.9% who would not take a CBCT scan in a pregnant patient added comments, such as ‘risk of complaint/litigation’, ‘patient may blame me for any neonatal issues’. The fear of litigation is a common reason for avoiding dental X‐ray imaging (Patil et al., 2013; Shrout et al., 1994; Strafford et al., 2008). More education is required to provide all stakeholders with the risks/benefits of exposing patients who are potentially more radiosensitive. The Faculty of General Dental Practitioners, Royal College of Surgeons (England) guidance document, Guidance Notes for Dental Practitioners on the Safe Use of X‐ray Equipment (Faculty of General Dental Practitioners, 2020) states that asking the patient about their pregnancy status is not normally relevant because the primary X‐ray beam should not irradiate the pelvic area (Faculty of General Dental Practitioners, 2020).

## CONCLUSION

CBCT is commonly used amongst UK endodontists. Most respondents used CBCT in their practice (86.1%). The most common uses related to complex root canal anatomy (89.1%), assessment and management of root resorption (87.4%), and pre‐operative assessment of periradicular surgery (84.5%). Respondents felt that CBCT improved confidence in their treatment planning (94.8%) and improved the quality of care (97%) they could offer their patients. More awareness of the legislation on reporting on medical exposures is required as 22.4% of clinicians did not report on their scans.

## AUTHOR CONTRIBUTIONS

S Patel: conceptualization, methodology, visualization, resources, writing – original draft, writing – review and editing, project administration. J Fitton writing, methodology, resources, review and editing. J Brown, F Foschi, N Al‐Nuaimi: writing – review and editing.

## CONFLICT OF INTEREST STATEMENT

The authors declare no conflict of interest.

## Data Availability

The data that support the findings of this study are available from the corresponding author upon reasonable request.
